# Identification of Circulating lncRNAs Associated with Gallbladder Cancer Risk by Tissue-Based Preselection, Cis-eQTL Validation, and Analysis of Association with Genotype-Based Expression

**DOI:** 10.3390/cancers14030634

**Published:** 2022-01-27

**Authors:** Alice Blandino, Dominique Scherer, Trine B. Rounge, Sinan U. Umu, Felix Boekstegers, Carol Barahona Ponce, Katherine Marcelain, Valentina Gárate-Calderón, Melanie Waldenberger, Erik Morales, Armando Rojas, César Munoz, Javier Retamales, Gonzalo de Toro, Olga Barajas, María Teresa Rivera, Analía Cortés, Denisse Loader, Javiera Saavedra, Lorena Gutiérrez, Alejandro Ortega, Maria Enriqueta Bertrán, Fernando Gabler, Mónica Campos, Juan Alvarado, Fabrizio Moisán, Loreto Spencer, Bruno Nervi, Daniel E. Carvajal-Hausdorf, Héctor Losada, Mauricio Almau, Plinio Fernández, Ivan Gallegos, Jordi Olloquequi, Macarena Fuentes-Guajardo, Rolando Gonzalez-Jose, Maria Cátira Bortolini, Carla Gallo, Andres Ruiz Linares, Francisco Rothhammer, Justo Lorenzo Bermejo

**Affiliations:** 1 Statistical Genetics Research Group, Institute of Medical Biometry, Heidelberg University, 69120 Heidelberg, Germany; blandino@imbi.uni-heidelberg.de (A.B.); scherer@imbi.uni-heidelberg.de (D.S.); boekstegers@imbi.uni-heidelberg.de (F.B.); barahona@imbi.uni-heidelberg.de (C.B.P.); garate@imbi.uni-heidelberg.de (V.G.-C.); 2 Department of Research, Cancer Registry of Norway, 0379 Oslo, Norway; trro@kreftregisteret.no (T.B.R.); sium@kreftregisteret.no (S.U.U.); 3 Department of Informatics, University of Oslo, 0304 Oslo, Norway; 4 Department of Basic and Clinical Oncology, Medical Faculty, University of Chile, Santiago 8380000, Chile; kmarcelain@uchile.cl (K.M.); olbeba@gmail.com (O.B.); gallegos@hcuch.cl (I.G.); 5 Research Unit Molecular Epidemiology and Institute of Epidemiology, Helmholtz Zentrum München, German Research Center for Environmental Health, 85764 Neuherberg, Germany; waldenberger@helmholtz-muenchen.de; 6 Hospital Regional de Talca, Talca 3460000, Chile; emoralesm@hospitaldetalca.cl (E.M.); cmunozc@hospitaldetalca.cl (C.M.); 7 Facultad de Medicina, Universidad Católica del Maule, Talca 3460000, Chile; arojasr@ucm.cl; 8 Instituto Nacional del Cáncer, Santiago 7500650, Chile; jretamales@gocchi.org; 9 Hospital de Puerto Montt, Puerto Montt 5480000, Chile; gonzalo.detoro@uach.cl; 10 Escuela de Tecnología Médica, Universidad Austral de Chile sede Puerto Montt, Puerto Montt 5480000, Chile; 11 Hospital Clínico Universidad de Chile, Santiago 8380456, Chile; 12 Hospital del Salvador, Santiago 7500922, Chile; memerivera@yahoo.es (M.T.R.); acortes@hsalvador.cl (A.C.); 13 Hospital Padre Hurtado, Santiago 8880456, Chile; denisseloader@gmail.com (D.L.); javierasaavedranazer@gmail.com (J.S.); 14 Hospital San Juan de Dios, Santiago, 8320000, Chile; lorenagutierrezc@yahoo.es; 15 Hospital Regional, Arica 1000000, Chile; alejandro.ortega@hjnc.cl; 16 Unidad Registro hospitalario de Cáncer, Hospital Base Valdivia, Valdivia 5090146, Chile; enriqueta.bertran@redsalud.gov.cl; 17 Hospital San Borja Arriarán, Santiago 8320000, Chile; gablerf@gmail.com (F.G.); moni.campos.m@gmail.com (M.C.); 18 Hospital Regional Guillermo Grant Benavente, Concepcion 4070386, Chile; jalvari@gmail.com (J.A.); fabriziomoisan@udec.cl (F.M.); loretospencer@gmail.com (L.S.); 19 Departamento de Hematología y Oncología, Escuela de Medicina, Pontificia Universidad Católica de Chile, Santiago 8330077, Chile; bnervi@gmail.com or; 20 Facultad de Medicina, Clínica Alemana Universidad del Desarrollo, Santiago 7650568, Chile; dcarvajal@alemana.cl; 21 Hospital de Temuco, Temuco 4780000, Chile; hector.losada@ufrontera.cl; 22 Hospital de Rancagua, Rancagua 2820000, Chile; mauricio.almau@gmail.com (M.A.); pliniofernandezbruno@gmail.com (P.F.); 23 Department of Biochemistry and Physiology, Faculty of Pharmacy and Food Sciences, University of Barcelona, 08028 Barcelona, Spain; jordiolloquequi@ub.edu; 24 Facultad de Ciencias de la Salud, Universidad Autónoma de Chile, Talca 3460000, Chile; 25 Departamento de Tecnología Médica, Facultad de Ciencias de la Salud, Tarapacá University, Arica 1000815, Chile; mafuentesg@academicos.uta.cl; 26 Instituto Patagónico de Ciencias Sociales y Humanas, Centro Nacional Patagónico, CONICET, Puerto Madryn U9120ACD, Argentina; rolando@cenpat-conicet.gob.ar; 27 Instituto de Biociências, Universidad Federal do Rio Grande do Sul, Puerto Alegre 15053, Brazil; maria.bortolini@ufrgs.br; 28 Laboratorios de Investigación y Desarrollo, Facultad de Ciencias y Filosofía, Universidad Peruana Cayetano Heredia, Lima 15102, Peru; carla.gallo@upch.pe; 29 Ministry of Education Key Laboratory of Contemporary Anthropology, Collaborative Innovation Center of Genetics and Development, School of Life Sciences and Human Phenome Institute, Fudan University, Shanghai 200434, China; a.ruizlin@ucl.ac.uk; 30 ADES (Anthropologie Bio-Culturelle, Droit, Éthique et Santé), UFR de Médecine, Aix-Marseille University, 13007 Marseille, France; 31 Department of Genetics, Evolution and Environment, UCL Genetics Institute, University College London, London WC1E 6BT, UK; 32 Instituto de Alta Investigación, Tarapacá University, Arica 1000000, Chile; franciscorothhammer@gmail.com

**Keywords:** gallbladder cancer, lncRNAs, eQTLs, genetic association study, molecular phenotypes

## Abstract

**Simple Summary:**

Gallbladder cancer (*GBC*) is an aggressive disease with poor prognosis that urgently needs risk biomarkers for prevention. Long noncoding RNAs (lncRNAs) have been linked to various types of cancer and have good potential as circulating biomarkers. Prediction of lncRNA expression based on genotype data may contribute to quantify individual *GBC* risk even without direct lncRNA expression measurement. In this study, we investigate the relationship between *GBC* risk and genotype-based expression of circulating lncRNAs.

**Abstract:**

Long noncoding RNAs (lncRNAs) play key roles in cell processes and are good candidates for cancer risk prediction. Few studies have investigated the association between individual genotypes and lncRNA expression. Here we integrate three separate datasets with information on lncRNA expression only, both lncRNA expression and genotype, and genotype information only to identify circulating lncRNAs associated with the risk of gallbladder cancer (*GBC*) using robust linear and logistic regression techniques. In the first dataset, we preselect lncRNAs based on expression changes along the sequence “gallstones → dysplasia → *GBC*”. In the second dataset, we validate associations between genetic variants and serum expression levels of the preselected lncRNAs (cis-lncRNA-eQTLs) and build lncRNA expression prediction models. In the third dataset, we predict serum lncRNA expression based on individual genotypes and assess the association between genotype-based expression and *GBC* risk. AC084082.3 and LINC00662 showed increasing expression levels (*p*-value = 0.009), while C22orf34 expression decreased in the sequence from gallstones to *GBC* (*p*-value = 0.04). We identified and validated two cis-LINC00662-eQTLs (r^2^ = 0.26) and three cis-C22orf34-eQTLs (r^2^ = 0.24). Only LINC00662 showed a genotyped-based serum expression associated with *GBC* risk (OR = 1.25 per log2 expression unit, 95% CI 1.04–1.52, *p*-value = 0.02). Our results suggest that preselection of lncRNAs based on tissue samples and exploitation of cis-lncRNA-eQTLs may facilitate the identification of circulating noncoding RNAs linked to cancer risk.

## 1. Introduction

Gallbladder cancer (*GBC*; International Classification of Diseases, 10th Revision, diagnosis code C23) is an aggressive malignancy responsible for around 85,000 deaths each year worldwide [1]. *GBC* early symptoms are unspecific, and less than 20% of patients are candidates for curative surgery at diagnosis. This translates into 5-year survival rates of 5% to 30%, depending on the country at diagnosis [2,3,4,5]. *GBC* incidence and mortality vary widely around the world, with about 65% of cases occurring in less developed countries [6]. Risk factors include the presence of gallstones (GS), female sex, high body mass index, and Native American ancestry [7,8]. As *GBC* develops over 10–20 years, generally following the sequence of gallstones “GS → dysplasia (Dys) → *GBC*”, there is ample opportunity for prevention [9].

Despite the large potential for primary prevention and early *GBC* diagnosis, especially considering the possibility of prophylactic surgical removal of the gallbladder (cholecystectomy), few studies have been conducted to identify *GBC* risk biomarkers.

Long noncoding RNAs (lncRNAs) are transcripts of more than 200 nucleotides that are not translated into proteins [10]. More and more studies are reporting that lncRNAs play crucial roles in the regulation of gene transcription, post-transcriptional and translational processes, and epigenetic modifications [11]. Altered lncRNA expression has been shown to be tightly correlated with the risk of multiple diseases, including cancer, and lncRNAs may have good potential to serve as biomarkers for risk prediction and therapeutic intervention [12,13,14].

The expression of particular lncRNAs seems to depend on the individual genotype to a certain extent. Single-nucleotide polymorphisms (SNPs) that modulate the expression of molecular phenotypes are denominated expression quantitative trait loci (eQTL). They may modulate the expression of chromosomally close (cis-eQTL) or distant transcripts (trans-eQTL). Recently, an increasing number of studies have attempted to infer mRNA expression based on genomewide SNPs, but the prediction of lncRNA expression relying on individual genotypes is still at a very early stage [15,16,17].

In the present study, which is based on three independent Chilean datasets—Chile shows one of highest *GBC* mortalities worldwide—we apply a three-stage approach to identify circulating lncRNAs as *GBC* risk biomarkers that may inform current prevention programs. We first preselect lncRNAs based on expression changes in gallbladder tissue along the sequence “GS → Dys → *GBC*”. Then, we identify and validate lncRNA-eQTLs in a second dataset. We finally predict the expression levels of circulating lncRNAs in a third independent dataset and estimate the association between genotype-based lncRNA expression and *GBC* risk.

## 2. Materials and Methods

**Figure 1:**
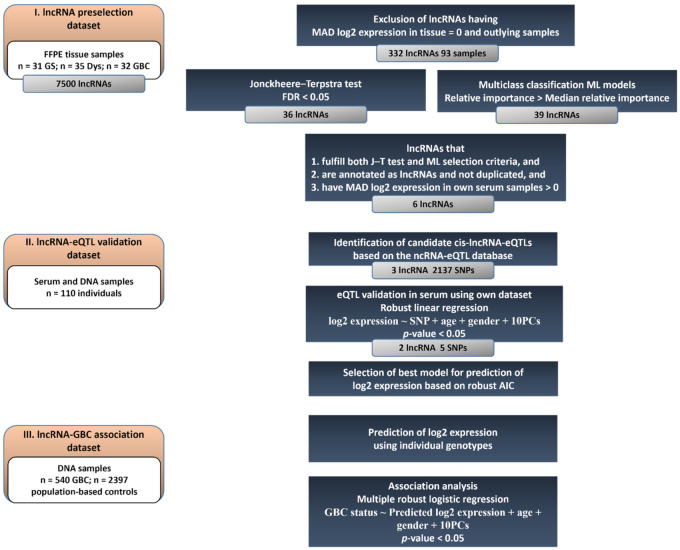
Flowchart representing the three-stage approach used in the study.

To identify circulating lncRNAs associated with *GBC* risk, we applied a three-stage approach that integrated three separated datasets with different information on lncRNA expression and individual genotypes. The first dataset (lncRNA preselection dataset) included only data on lncRNA expression that was used to identify lncRNAs with monotonically increasing or decreasing expression levels in gallbladder tissue along the model of *GBC* development “GS → Dys → *GBC*”. A second, independent dataset that included both lncRNA expression and genotype data (lncRNA-eQTL validation dataset) was used to identify and validate genetic variants associated with the expression of the preselected lncRNAs in serum (cis-lncRNA-eQTLs). Finally, the genotype-based expression in serum was predicted in a third dataset with individual genotype information only (lncRNA-*GBC* association dataset), and the association between *GBC* risk and predicted lncRNA serum expression was quantified. Figure 1 represents the datasets used and the methods applied in the present study.

### 2.1. RNA Extraction and Small RNA Sequencing

Formalin-fixed, paraffin-embedded (FFPE) gallbladder tissue specimens were obtained from 98 patients in total (*n* = 31 GS; *n* = 35 Dys; *n* = 32 *GBC*). RNA was extracted from FFPE sections using the AllPrep FFPE kit following Qiagen’s recommendations, and RNA quality was controlled (High Sensitivity Genomic DNA, Advanced Analytical, United States, and FFPE quality control kits, Illumina).

The NEBNext Small RNA kit (NEB) was used to produce RNA sequencing libraries, which were sequenced on the HiSeq 2500 platform (Illumina, San Diego, CA, USA) to an average depth of 18 M reads per sample. The applied RNA sequencing protocol has been previously described in detail [18]. Briefly, our protocol enabled us to capture lncRNA mapped fragments in the size range up to 47 base pairs. First, reads from the HiSeq 2500 platform were adapter-trimmed (AdapterRemoval v2.1.7) [19]. Then, adapter-trimmed reads were mapped to the human genome (hg38) by a Bowtie2 v2.2.9 aligner [20]. HTSeq was used to count reads mapped to lncRNA regions in GENCODE v26 annotations [21,22].

### 2.2. DNA Extraction and Genotyping

Genomic DNA was extracted from peripheral blood or saliva using standard commercial kits and following standard laboratory procedures. Intraplate and interplate replicates and blinded duplicates were included (at 5%) as quality control measures. Study participants were genotyped with Illumina’s OmniExpress or Global Screening arrays. Both arrays included more than 700,000 genomewide SNPs. Genotypes were imputed with the minimac4 imputation software and the TOPMed reference sample via the TOPMed imputation server, accessible at https://imputation.biodatacatalyst.nhlbi.nih.gov/ (accessed on 1 August 2021) [23].

### 2.3. Patients and Statistical Analyses for lncRNA Preselection

Chilean patients with GS (those who underwent cholecystectomy without *GBC* findings), Dys, and *GBC* were invited to participate. Except for two patients with *GBC* and missing GS information, all the patients with *GBC* and Dys in the study carried GS. Upon written informed consent, the patients were interviewed by the study coordinators, who retrieved tissue samples and clinical information using standardized case report forms. Samples stored for >5 years and patients with porcelain gallbladder, polyps, noncholesterol stones, or pancreatic/bile duct abnormalities were excluded. This cohort of patients has previously been described in detail [24].

Read counts were transformed to log2 transcripts per million. Log2 expression values with low variability (median absolute deviation (MAD) = 0) were excluded from subsequent statistical analyses. Quantile normalization was first applied to GS, Dys, and *GBC* expression values separately, and then to the complete dataset. Principal component analysis (PCA) was performed for an unsupervised examination of the global expression profiles and identification of potential patients with outlying expression profiles. After PCA, the Mahalanobis depth (MD) was calculated, and 5% of the samples with the lowest MD were excluded. The R package “stats” was used for PCA and MD calculation [25].

LncRNA preselection relied on both nonparametric and machine learning (ML) techniques, which were simultaneously performed to improve the robustness of our findings. Nonparametric two-sided Jonckheere–Terpstra (J–T) tests with *n* = 5000 permutations were conducted to identify lncRNAs with monotonically increasing or decreasing expression levels in gallbladder tissue along the model of *GBC* development “GS → Dys → *GBC*” using the “JonckheereTerpstraTest” function of the R package “DescTools” [26]. Multiplicity-corrected *p*-values were transformed into false discovery rates (FDRs).

The extreme gradient boosting (XGBoost) algorithm was used to train three-class classification ML models. We utilized the R implementation (v3.5.3) of this algorithm in the h2o R package (v3.32.1.5) [27]. A complete dataset was randomly separated into training (*n* = 77) and test (*n* = 21) sets. The classes were balanced in the training dataset by upsampling, resulting in 27 GS, Dys, and *GBC* samples per group. Fivefold cross validation was utilized to tune hyperparameters of the model using only the training dataset. A random grid search approach was applied. After cross validation, the best model with the lowest mean per class error was selected. Then, the best model’s performance was measured on the test dataset using both mean per class error and area under the ROC curve (AUC) for multinomial models (i.e., weighted average AUC). Relative importance values were extracted using the function “h2o.varimp”. Model parameters, R code, and seed values are provided in the Supplementary Materials.

Figure 1 depicts the criteria applied to preselect the lncRNAs, which included: (i) J–T FDR < 0.05 and relative importance higher than the median, (ii) they were annotated as lncRNAs and were not duplicated, (iii) nonzero MAD log2 expression in the lncRNA-eQTL validation dataset, and (iv) information available in the ncRNA-eQTL database.

### 2.4. Individuals and Statistical Analyses for lncRNA-eQTL Validation

The dataset used for the identification and validation of cis-lncRNA-eQTLs included both genomewide genotype and serum lncRNA expression data for 110 participants in two Chilean studies on chronic obstructive pulmonary disease (COPD, *n* = 22) and Chagas disease (*n* = 88). Information on GS and cancer history was not available, but the incidence of GS and cancer in the two studies should be representative of the general Chilean population.

A preliminary list of cis-lncRNA-eQTLs potentially associated with our preselected candidates was obtained from the ncRNA-eQTL database: http://ibi.hzau.edu.cn/ncRNA-eQTL/ (accessed on 1 August 2021).

LncRNA read counts were log2-transformed and quantile-normalized. Genetic variants were filtered to exclude SNPs with a missing call rate higher than 5% or a minor allele frequency (MAF) below 1%. Samples with a missing call rate over 5% were also filtered out. Identity by descent (IBD) kinship coefficients between pairs of individuals were calculated, and individuals within each related pair (IBD > 0.1) with the lowest call rate were consequently eliminated. After linkage disequilibrium (LD) pruning at r^2^ > 0.1, 36,175 variants from the GSA array were used for the subsequent genetic PCA, and MDs were calculated to exclude participants with departing genotypes (5% of individuals with the lowest statistical depth). MAF and call rates were calculated using the R functions “col.summary” and “row.summary” available at Bioconductor’s package “snpStats” [28]. The R package “SNPRelate” was used to calculate IBD kinship coefficients and perform LD pruning (functions: “snpgdsIBDMoM”, “snpgdsLDpruning”) [29]. Genetic PCA was conducted using the eigenstrat function available at: www.popgen.dk/software/index.php/Rscripts (accessed on 1 August 2021).

Cis-lncRNA-eQTL associations found in the ncRNA-eQTL database were validated using our own lncRNA-eQTL validation dataset. Robust linear regression models were fitted considering the individual age and gender and the first 10 genetic *PCs*:

(1) $$\log_{2}\;expression\;\sim \;SNP+age+gender+10PCs$$

Four penetrance models were investigated for each genetic variant in the linear regression models: Additive (count of major alleles), Three-Genotype (genotype as a categorical variable), Dominant (at least one affect allele vs. the other genotype), Recessive (two affect alleles vs. the other genotypes).

After considering genetic variants separately, we included combinations of the identified cis-lncRNA-eQTLs in the fitted robust linear regression models in addition to age, gender, and the first 10 *PCs*. The model with the lowest robust Akaike’s information criterion (RAIC) was selected for subsequent prediction of log2 expression levels in serum.

Robust linear regression models were fitted using the function “rlm” in the R package “MASS” [30]. The corresponding *p*-values were obtained using the function “rob.pvals” from the R package “clickR” [31]. RAIC for each model was calculated using the function “AIC” in the R package “AICcmodavg” [32].

### 2.5. Patients and Population-Based Controls and Statistical Analyses on the Association between Genotype-Based lncRNA Expression and GBC Risk

Serum lncRNA expression was predicted based on individual genotype data from 540 Chilean *GBC* patients and 2397 population-based controls. *GBC* patients were recruited between 2014 and 2020. Except for a few patients who were diagnosed without undergoing cholecystectomy, the majority of the *GBC* patients (77%) were diagnosed after surgical removal of the gallbladder. Population-based controls were selected from the Chilean subset of the Consortium for the Analysis of the Diversity and Evolution of Latin America (CANDELA) and from Chilean studies on COPD and Chagas disease with GS and cancer incidences representative of the general Chilean population [7,8,33].

Individual lncRNA log2 serum expression levels were predicted considering the effect estimates from the linear robust regression models fitted to the lncRNA-eQTL validation dataset (*βi*) and the individual genotype (*Ai*) encoded according to the selected penetrance model:

(2) $$Predicted\;log2\;serum\;expression=\overset{k}{\underset{i=1}{\sum }}\beta_{i}A_{i}\;$$

Note that, due to the discrete nature of individual genotypes, predicted expression levels are also discrete.

Finally, the association between genotype-based serum lncRNA expression and *GBC* risk was assessed by robust logistic regression models using a tuning constant c in Huber’s psi-function equal to 1.2, considering the individual age and gender, and the first 10 genetic *PCs*:

(3) GBC status ~ Predicted log2 serum expression+age+gender+10PCs 

Robust logistic regression models were fitted using the function “glmrob” from the R package “robustbase” [34]. Plots were generated using the R package “ggplot2” [35]. Analyses were all conducted in R, version 4.0.3.

## 3. Results

### 3.1. Preselected lncRNAs

**Figure 2:**
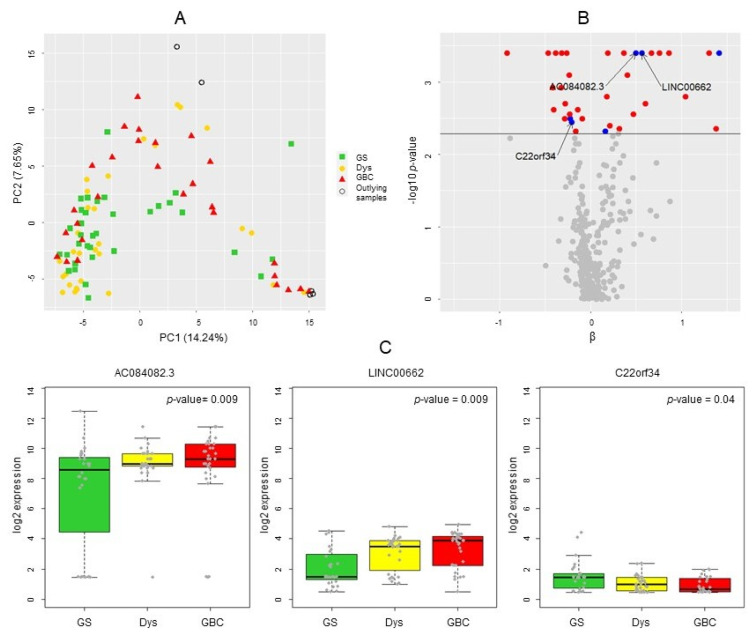
LncRNA preselection. (**A**) PCA based on normalized log2 expression counts for lncRNAs with a nonzero MAD expression in the preselection dataset. (**B**) Volcano plot for the lncRNAs with nonzero MAD expression investigated in the lncRNA preselection dataset. The *y*-axis shows −log10 *p*-values from J–T tests. The black line represents the applied threshold (FDR = 0.05). The red dots highlight lncRNAs preselected according to both J–T tests and ML, which showed low expression variability (MAD = 0) in serum samples. The blue dots show the six candidates that fulfilled both J–T and ML preselection criteria, with nonzero MAD expression in serum samples. (**C**) Dot-and-box plots of log2 expression in GS, Dys, and *GBC* tissue samples for the three preselected lncRNAs.

We detected a total of 7500 lncRNAs in the preselection dataset. Among them, 7168 lncRNAs showed a MAD of 0 and were excluded. PCA results considering the remaining 332 lncRNAs are shown in Figure 2A. Five individuals with the lowest statistical depth consistent with outlying global expression profiles were also excluded. The final preselection dataset comprised 332 lncRNAs and 93 samples (*n* = 28 GS, *n* = 34 Dys, *n* = 31 *GBC*).

Multiplicity-corrected *p*-values from two-sided J–T tests identified 36 lncRNAs with monotonically increasing or decreasing expression levels (FDR < 0.05) along the sequence “GS → Dys → *GBC*” (Figure 2B, Table S1).

The ML model separated between GS, Dys, and *GBC* with an AUC of 0.88 and a mean per class error of 0.23. The best model selected 76 lncRNAs as class predictors. Among them, 39 lncRNAs with relative importance higher than the median were selected (Figure S1).

Eighteen lncRNAs fulfilled both nonparametric J–T test and ML selection criteria. All were annotated as lncRNAs, and none was duplicated. Six out of the 18 lncRNAs showed a nonzero MAD log2 expression in serum samples from the cis-lncRNA-eQTL validation dataset. Among them, 3 lncRNAs (AC084082.3, LINC00662, and C22orf34) were found in the ncRNA-eQTL database and consequently fulfilled all the preselection criteria for subsequent lncRNA-eQTL validation (Figure 1). The expression of AC084082.3 and LINC00662 monotonically increased with advancing malignancy, while the expression level of C22orf34 decreased in the sequence from GS to *GBC* (Figure 2C).

Table 1 shows the expression of AC084082.3, LINC00662, and C22orf34lnc in GS, Dys, and *GBC* tissue samples. With the exception of LINC00662, larger average expression differences were found between GS and *GBC* than between GS and Dys. As expected, the investigated patients included more women than men. Age-stratified analyses revealed larger expression differences for LINC00662 in younger patients and larger expression differences for C22orf34lnc in older patients, although the differences in differences did not reach statistical significance (overlapping 95% confidence intervals).

### 3.2. Validated lncRNA-eQTLs

**Figure 3:**
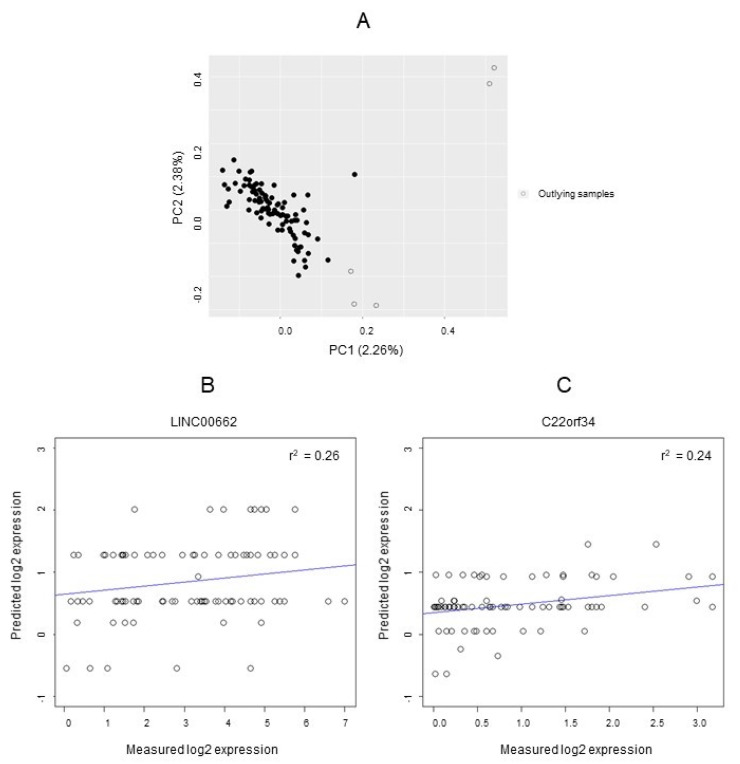
LncRNA-eQTL validation. (**A**) Genetic PCA based on LD-pruned genotypes from the lncRNA-eQTL validation dataset. (**B**,**C**) Measured vs. predicted log2 expression for LINC00662 and C22orf34, respectively.

In the lncRNA-eQTL validation dataset, 460,632 SNPs with low MAF, 4 individuals with a low call rate, and 8 related individuals (IBD coefficient > 0.1) were excluded. Figure 3A shows the results from the genetic PCA. After exclusion of 5 outlying individuals with the lowest statistical depth, the final dataset included 93 individuals.

According to the ncRNA-eQTL database, 161 cis-lncRNA-eQTLs were associated with AC084082.3 expression. Ten of them were excluded due to a low MAF or call rate, and robust linear regression did not identify any association with the expression of AC084082.3 in the lncRNA-eQTL validation dataset considering the four investigated penetrance models.

Among the 1576 cis-lncRNA-eQTLs associated with LINC00662 expression according to the ncRNA-eQTL database, 1388 SNPs were available in the lncRNA-eQTL validation dataset, fulfilled quality control criteria, and were retained for subsequent analyses. Robust linear regression identified 2 cis-LINC00662-eQTLs: rs11083486 (associated with the four penetrance models) and rs142521755 (dominant association). Rs11083486 and rs142521755 are not in LD (r^2^ = 0.001), which indicates independent associations. The best model to predict LINC00662 expression (lowest RAIC = 357) included rs11083486 (additive penetrance) and rs142521755 (dominant penetrance). We examined the relative relevance of the SNPs for the prediction of LINC00662 expression by comparing the coefficient of multiple determination (r^2^) for the selected full regression model versus a reference model that only included age, gender, and the first 10 *PCs*. The proportion of variance in LINC00662 expression explained by the full regression model was r^2^ = 0.26, compared with r^2^ = 0.17 for the reference model.

A total of 396 cis-lncRNA-eQTLs were associated with C22orf34 expression according to the ncRNA-eQTL database, but 18 SNPs did not fulfill quality control criteria. We reproduced the association between 45 SNPs and C22orf34 expression in the lncRNA-eQTL validation dataset. A total of 42 SNPs were excluded after LD pruning, resulting in 3 cis-C22orf34-eQTLs: rs5770650 and rs9628049 (both associated with the additive and dominant models) and rs6009824 (three-genotype model). The best model for C22orf34 prediction (lowest RAIC = 214.5) included rs5770650 (additive penetrance), rs9628049 (additive penetrance), and rs6009824 (three-genotype). Additionally, for the prediction of C22orf34 expression, the proportion of variance explained by the full regression model was r^2^ = 0.24, compared with r^2^ = 0.06 for the reference model without cis-C22orf34-eQTLs.

Panels B and C in Figure 3 compare the measured log2 expression with the genotype-based expression of LINC00662 and C22orf34, respectively. All identified cis-lncRNA-eQTLs are shown in Table S2, and the validated cis-lncRNA-eQTLs are shown in Table 2.

### 3.3. LncRNAs with Genotype-Based Plasma Expression Associated with GBC Risk

**Figure 4:**
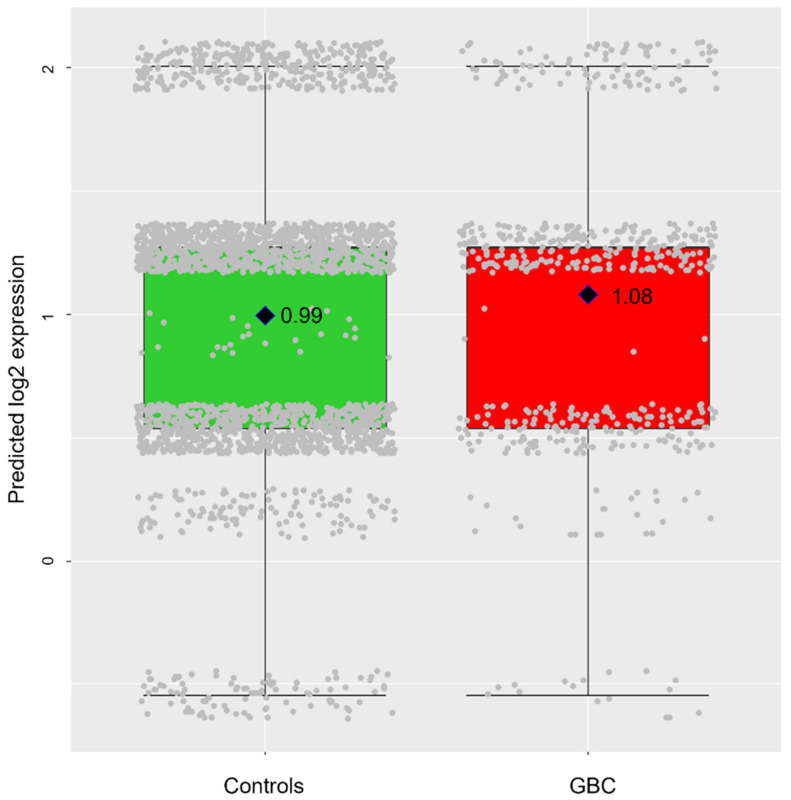
Predicted genotype-based log2 LINC00662 expression in the lncRNA-*GBC* association dataset. Rhombuses represent the average genotype-based log2 expression in population-based controls and *GBC* patients.

The final goal of this study was the identification of circulating lncRNAs that may serve as biomarkers for *GBC* risk prediction. We thus investigated the association between predicted genotype-based lncRNA expression levels and *GBC* risk for LINC00662 and C22orf34 in an independent dataset with 540 *GBC* patients and 2397 population-based controls (lncRNA-*GBC* association dataset, Figure 1). Six expression levels were predicted for LINC00662 (additive model 3 categories × dominant model 2 categories) and 10 levels for C22orf34 (additive model × additive model × three-genotype), but not all categories were represented (Figure 4 and Figure S2).

In agreement with expression measurements in gallbladder tissue, genotype-based expression of LINC00662 in serum was higher in *GBC* patients than in population-based controls, translating into a 25% increased risk of *GBC* per log2 expression unit (OR = 1.25, 95% CI = 1.04–1.52, *p*-value = 0.02, Table 3 and Figure 4).

The genotype-based expression of C22orf34 was lower in *GBC* patients than in population-based controls, but the *GBC* risk increase did not reach statistical significance (OR = 0.90, 95% CI = 0.61–1.32, *p*-value = 0.59, Table 3 and Figure S2).

## 4. Discussion

In the present study, we aimed at the identification of circulating lncRNAs as potential biomarkers for *GBC* prevention utilizing genotype-based lncRNA expression levels. *GBC* is relatively rare in high-income countries, but common in several low- and middle-income countries and extremely aggressive. The disease develops over the course of 10 to 20 years, facilitating the implementation of primary and secondary personalized prevention strategies. Individual estimates of *GBC* risk would guide surveillance and aid personal decisions on the possible benefit of prophylactic cholecystectomy for persons at high risk (e.g., first-degree relatives of *GBC* patients, severely obese women, and patients with large GS). A reduction in the number of unnecessary cholecystectomies, while simultaneously detecting *GBC* with high sensitivity, would be particularly relevant in low-income regions with high *GBC* incidences and limited financial and clinical resources.

We thus applied a multistage approach through a combination of three independent Chilean datasets with information on (1) lncRNA expression only, (2) lncRNA expression and genotype information, and (3) genotype information only. Using both nonparametric (J–T test) and ML (XGBoost algorithm) techniques, we preselected three lncRNAs that showed gradual changes in tissue expression along the sequence of GS, Dys, and *GBC*. AC084082.3 and LINC00662 showed increasing expression levels with advancing malignancy, while the expression of C22orf34 decreased along the sequence from GS to *GBC*. Then, we were able to identify and validate two cis-LINC00662-eQTLs and three cis-C22orf34-eQTLs. Finally, in our last independent dataset with genotype information only, we predicted the expression of LINC00662 and C22orf34 relying on individual genotypes. Results from robust logistic regression revealed an association between the genotype-based expression in serum of LINC00662 and *GBC* risk.

The use of lncRNAs as biomarkers for predicting *GBC* holds great potential, as lncRNA expression has been shown to play an important role in tumorigenesis and metastasis of many human cancers [12,13,14]. Moreover, lncRNAs are highly stable in serum even under extreme temperature and pH conditions and long-term storage. Therefore, they are good candidates for predicting *GBC* risk and preventing *GBC* in low-income regions.

Whereas, to our knowledge, the roles of AC084082.3 and C22orf34 in tumors have not been reported in the literature to date, several studies indicate that the preselected candidate LINC00662, which showed a genotype-based expression in serum associated with *GBC* risk, might be a promising biomarker for cancer diagnosis and therapy. LINC00662 was first reported to be highly expressed in patients with lung squamous cell carcinoma [36]. Another study on lung cancer highlighted that the expression of LINC00662 promotes cell invasion and contributes to cancer stem cell-like phenotypes in lung cancer cells [37]. Bioinformatics analysis in gastric cancer suggested that LINC00662 overexpression is tightly related to poor patients’ prognosis [38]. Furthermore, overexpression of LINC00662 has also been observed in other types of tumors, including breast, cervical, and prostate cancers and chordoma, glioma, and hepatocellular carcinoma [39]. LINC00662 has been shown to participate in regulating mRNA stability as a mediator of gene expression, and to participate in different signaling pathways [39]. Unfortunately, the ncRNA-eQTL database does not include specific information on lncRNA-eQTLs for *GBC*; however, some of our validated lncRNA-eQTLs are linked to other cancer types [40]. Interestingly, the association between the expression of LINC00662 and rs11083486 was also observed in patients with bladder carcinoma, whereas rs5770650 was found to be associated with C22orf34 expression in hepatocellular carcinoma.

Overall, our results confirm that exploiting individual genotype data to predict ncRNA expression has good potential. The novelty of our approach relies on the combination of three independent datasets, in which we performed (1) lncRNA candidate preselection, (2) cis-lncRNA-eQTL validation, and (3) association analysis between genotype-based lncRNA expression and *GBC* risk. Instead of using standard statistical methods to detect differentially expressed lncRNAs, we combined nonparametric and ML techniques and considered only lncRNAs preselected through both methods. Adjustment for potential confounders and a population substructure in the lncRNA-eQTL stage represented another strength of our study. The potential of our approach is also demonstrated by the association found in the prediction stage. Only the genotype-based expression of LINC00662 was associated with *GBC* risk, and the consistent results in the preselection stage (based on FFPE tissue samples) and prediction stage (based on individual genotypes) add plausibility to our findings.

The small sample size of the preselection and cis-lncRNA-eQTL validation datasets was a limitation of our study. With a larger number of patients, we probably could have validated more associations and possibly preselected more lncRNA candidates. The low number of validated associations compared with those identified in the ncRNA-eQTL database may also be related to differences in genetic background between the Chilean individuals in our three datasets and the investigated patients in the ncRNA-eQTL database. In addition, molecular and genetic differences between datasets can translate into inability to validate some promising candidates. For example, many of the preselected lncRNAs showed a highly variable expression in FFPE tissue but low variability in serum samples and were therefore excluded from subsequent analyses. A limitation, but also a strength, of the present study was the directionality of the associations investigated. Predicting lncRNA expression based on individual genotypes allows the association “lncRNA → *GBC*” to be examined, and associations identified in this direction are particularly relevant for risk prediction and disease prevention. However, the reverse association “*GBC* → lncRNA” cannot be investigated using the approach described in this study.

## 5. Conclusions

*GBC* is relatively rare in high-income countries and understudied. Furthermore, genetic studies on molecular phenotypes are mostly based on individuals of European descent, and lncRNA and genotype data from Latin Americans are still limited. In this study, we aimed to identify risk biomarkers for *GBC* prevention in Chile, which has one of the highest *GBC* mortality rates in the world. We identified LINC00662 as a potential candidate, but the increased LINC00662 expression in serum samples from *GBC* patients needs to be validated in independent studies. In addition, it would be interesting to examine the potential of LINC00662 as a *GBC* risk biomarker in other world populations.

## Figures and Tables

**Table 1:** FFPE tissue expression of the three preselected lncRNAs in the complete dataset and stratified results by gender and age.

Subgroup	lncRNA	FDR *	log2 Expression in GS Samples Median (5th; 95th Percentiles)	log2 Expression Difference ^†^ Dys vs. GS Estimate (95% CI)	log2 Expression Difference ^†^ *GBC* vs. GS Estimate (95% CI)
All	AC084082.3	0.009	8.23 (1.45–9.93)	0.51 (0.04; 0.99)	0.76 (0.09; 1.44)
*n* = 28 GS; *n* = 34 Dys;	LINC00662	0.009	1.48 (0.55–4.38)	1.09 (0.62; 1.56)	0.86 (0.30; 1.42)
*n* = 31 *GBC*	C22orf34	0.04	1.44 (0.48–3.68)	−0.24 (−0.49; 0.005)	−0.28 (−0.54; −0.01)
Women	AC084082.3	0.04	8.23 (1.45–9.78)	0.67 (0.18; 1.15)	0.89 (0.15; 1.63)
*n* = 26 GS; *n* = 20 Dys;	LINC00662	0.01	1.47 (0.54–4.07)	1.09 (0.61; 1.56)	1.01 (0.45; 1.57)
*n* = 24 *GBC*	C22orf34	0.02	1.44 (0.48–3.80)	−0.30 (−0.57; −0.03)	−0.34 (−0.63; −0.04)
Men	AC084082.3	0.99	10.01	−0.52 (−1.02; −0.03)	−0.30 (−2.19; 1.59)
*n* = 1 GS; *n* = 8 Dys;	LINC00662	0.99	4.53	−0.52 (−1.24; 0.21)	−1.09 (−2.85; 0.68)
*n* = 6 *GBC*	C22orf34	0.99	0.49	0.43 (−0.66; 1.53)	0.27 (−0.19; 0.72)
Age < 60	AC084082.3	0.43	8.23 (1.45–10.19)	0.73 (0.13; 1.33)	0.64 (−0.22; 1.50)
*n* = 18 GS; *n* = 11 Dys;	LINC00662	0.51	1.81 (0.58–4.33)	0.93 (0.30; 1.55)	0.66 (−0.13; 1.45)
*n* = 9 *GBC*	C22orf34	0.58	1.43 (0.47–3.08)	−0.35 (−0.72; 0.02)	−0.29 (−0.67; 0.09)
Age > =60	AC084082.3	0.17	8.96 (1.47–9.86)	0.29 (−0.33; 0.90)	0.84 (−0.10; 1.77)
*n* = 9 GS; *n* = 16 Dys;	LINC00662	0.05	1.46 (0.78–3.84)	1.24 (0.67; 1.81)	1.06 (0.36; 1.77)
*n* = 18 *GBC*	C22orf34	0.17	1.46 (0.50–3.44)	−0.18 (−0.52; 0.16)	−0.34 (−0.68; 0.006)

* FDR: false discovery rate from two-sided Jonckheere–Terpstra test. Small *p*-values suggest monotonically increasing or decreasing expression levels. ^†^ Average log2 expression differences were estimated using robust linear regression.

**Table 2:** Identified and subsequently validated cis-lncRNA-eQTLs for the three preselected lncRNAs.

lncRNA	log2 Expression in Serum Median (5th; 95th Percentiles)	Chromosomal Location (GRCh38)	No. of Candidate cis-lncRNA-eQTLs in the ncRNA-eQTL Database	No. of Validated cis-lncRNA-eQTLs	No. of cis-lncRNA-eQTLs Used as Predictors	Adjusted r^2^ for the Best Prediction Model
AC084082.3	6.59 (1.74; 9.06)	chr8:66112667–66115207	161	-	-	-
LINC00662	3.40 (0.35; 5.60)	chr19:27684580–27793940	1576	2	2	0.26
C22orf34	0.58 (0.03; 2.65)	chr22:49414524–49657542	395	45	3	0.24

**Table 3:** Predicted genotype-based log2 expression of LINC00662 and C22orf34 and their association with *GBC* risk in the lncRNA-*GBC* association dataset.

lncRNA	Median Predicted log2 Expression	OR * (*GBC*)	95% CI ^†^	*p*-Value
LINC00662	1.27	1.25	1.04; 1.52	0.02
C22orf34	0.39	0.90	0.61; 1.32	0.59

* OR: odds ratio, adjusted by age and gender. ^†^ CI: confidence interval.

## Data Availability

The source code to reproduce all the results described is provided as Supplementary Material, and the necessary input files are available at www.biometrie.uni-heidelberg.de/StatisticalGenetics/Software_and_Data (accessed on 19 November 2021). The dataset used for the analyses described in this manuscript has been deposited in ArrayExpress at https://www.ebi.ac.uk/arrayexpress/ (accessed on 19 November 2021) with accession number E-MTAB-11367.

## Supplementary Materials

The following are available online at https://www.mdpi.com/article/10.3390/cancers14030634/s1: Figure S1: 39 preselected lncRNA candidates using ML, ordered by relative importance; Figure S2: Predicted log2 expression for C22orf34 in the lncRNA-*GBC* association dataset; Table S1: 36 preselected lncRNA candidates using J–T tests; Table S2: Identified and validated cis-lncRNA-eQTLs for the three preselected candidates.

## References

[1] Sung H., Ferlay J., Siegel R.L., Laversanne M., Soerjomataram I., Jemal A., Bray F. (2021). Global Cancer Statistics 2020: GLOBOCAN Estimates of Incidence and Mortality Worldwide for 36 Cancers in 185 Countries. CA Cancer J. Clin..

[2] Zhu X., Zhang X., Hu X., Ren H., Wu S., Wu J., Wu G., Si X., Wang B. (2020). Survival analysis of patients with primary gallbladder cancer from 2010 to 2015: A retrospective study based on SEER data. Medicine.

[3] Kanthan R., Senger J.-L., Ahmed S., Kanthan S.C. (2015). Gallbladder Cancer in the 21st Century. J. Oncol..

[4] Witjes C.D., Akker S.A.V.D., Visser O., Karim-Kos H.E., De Vries E., Ijzermans J.N., De Man R.A., Coebergh J.W.W., Verhoef C. (2012). Gallbladder Cancer in the Netherlands: Incidence, Treatment and Survival Patterns since 1989. Dig. Surg..

[5] Bertran E., Heise K., Andia M.E., Ferreccio C. (2010). Gallbladder cancer: Incidence and survival in a high-risk area of Chile. Int. J. Cancer.

[6] World Cancer Research Fund International, American Institute for Cancer Research (2015). Continuous Update Project Report: Diet, Nutrition, Physical Activity and Gallbladder Cancer.

[7] Barahona Ponce C., Scherer D., Brinster R., Boekstegers F., Marcelain K., Gárate-Calderón V., Müller B., de Toro G., Retamales J., Barajas O. (2020). Gallstones, Body Mass Index, C-reactive Protein and Gallbladder Cancer—Mendelian Randomiza-tion Analysis of Chilean and European Genotype Data. Hepatology.

[8] Bermejo J.L., Boekstegers F., Silos R.G., Marcelain K., Benavides P.B., Ponce C.B., Müller B., Ferreccio C., Koshiol J., Fischer C. (2017). Subtypes of Native American ancestry and leading causes of death: Mapuche ancestry-specific associations with gallbladder cancer risk in Chile. PLoS Genet..

[9] Wistuba I.I., Gazdar A.F. (2004). Gallbladder cancer: Lessons from a rare tumour. Nat. Cancer.

[10] Derrien T., Johnson R., Bussotti G., Tanzer A., Djebali S., Tilgner H., Guernec G., Martin D., Merkel A., Knowles D.G. (2012). The GENCODE v7 catalog of human long noncoding RNAs: Analysis of their gene structure, evolution, and expression. Genome Res..

[11] He R.-Z., Luo D.-X., Mo Y.-Y. (2019). Emerging roles of lncRNAs in the post-transcriptional regulation in cancer. Genes Dis..

[12] Fang Y., Fullwood M.J. (2016). Roles, Functions, and Mechanisms of Long Non-coding RNAs in Cancer. Genom. Proteom. Bioinform..

[13] Mercer T.R., Dinger M.E., Mattick J.S. (2009). Long non-coding RNAs: Insights into functions. Nat. Rev. Genet..

[14] Cesana M.C.D., Legnini I., Santini T., Sthandier O., Chinappi M., Tramontano A., Bozzoni I. (2011). A long noncoding RNA controls muscle differentiation by functioning as a competing endogenous RNA. Cell.

[15] Visscher P.M., Wray N.R., Zhang Q., Sklar P., McCarthy M.I., Brown M.A., Yang J. (2017). 10 years of GWAS Discovery: Biology, function, and translation. Am. J. Hum. Genet..

[16] Wu C., Miao X., Huang L., Che X., Jiang G., Yu D., Yang X., Cao G., Hu Z., Zhou Y. (2011). Genome-wide association study identifies five loci associated with susceptibility to pancreatic cancer in Chinese populations. Nat. Genet..

[17] Shastry B.S. (2009). SNPs: Impact on gene function and phenotype. Methods Mol. Biol..

[18] Umu S.U., Langseth H., Bucher-Johannessen C., Fromm B., Keller A., Meese E., Lauritzen M., Leithaug M., Lyle R., Rounge T.B. (2017). A comprehensive profile of circulating RNAs in human serum. RNA Biol..

[19] Schubert M., Lindgreen S., Orlando L. (2016). AdapterRemoval v2: Rapid adapter trimming, identification, and read merging. BMC Res. Notes.

[20] Langmead B., Salzberg S.L. (2012). Fast gapped-read alignment with Bowtie 2. Nat. Methods.

[21] Anders S., Pyl P.T., Huber W. (2015). HTSeq—A Python framework to work with high-throughput sequencing data. Bioinformatics.

[22] Harrow J., Frankish A., Gonzalez J.M., Tapanari E., Diekhans M., Kokocinski F., Aken B.L., Barrell D., Zadissa A., Searle S. (2012). GENCODE: The reference human genome annotation for The ENCODE Project. Genome Res..

[23] Taliun D., Harris D.N., Kessler M.D., Carlson J., Szpiech Z.A., Torres R., Taliun S.A.G., Corvelo A., Gogarten S.M., NHLBI Trans-Omics for Precision Medicine (TOPMed) Consortium (2021). Sequencing of 53,831 diverse genomes from the NHLBI TOPMed Program. Nature.

[24] Brägelmann J., Barahona Ponce C., Marcelain K., Roessler S., Goeppert B., Gallegos I., Colombo A., Sanhueza V., Morales E., Rivera M.T. (2020). Epigenome-wide analysis of methylation changes in the sequence of gallstone disease, dysplasia, and gallbladder cancer. Hepatology.

[25] R Core Team (2013). R: A Language and Environment for Statistical Computing.

[26] Signorell A., Aho K., Alfons A., Anderegg N., Aragon T., Arppe A. (2021). DescTools: Tools for Descriptive Statistics. R Package Version 0.99.44. https://cran.r-project.org/package=DescTools.

[27] Ledell E., Gill N., Aiello S., Fu A., Candel A., Click C., Kraljevic T., Nykodym T., Aboyoun P., Kurka M. (2022). R Interface for the ‘H_2_O’ Scalable Machine Learning Platform. R Package Version 3.36.0.1. https://CRAN.R-project.org/package=h2o.

[28] Clayton D. (2020). snpStats: SnpMatrix and XSnpMatrix Classes and Methods. R Package Version 1.40.0. https://bioconductor.org/packages/release/bioc/html/snpStats.html.

[29] Zheng X., Levine D., Shen J., Gogarten S.M., Laurie C., Weir B.S. (2012). A high-performance computing toolset for relatedness and principal component analysis of SNP data. Bioinformatics.

[30] Venables W.N., Ripley B.D. (2002). Modern Applied Statistics with S-PLUS.

[31] Marin D.H. (2021). clickR: Semi-Automatic Preprocessing of Messy Data with Change Tracking for Dataset Cleaning. R Package Version 0.8.0. https://CRAN.R-project.org/package=clickR.

[32] Marc J. (2020). Mazerolle AICcmodavg: Model Selection and Multimodel Inference Based on (Q)AIC(c). R Package Version 2.3-1. https://cran.r-project.org/package=AICcmodavg.

[33] Boekstegers F., Marcelain K., Ponce C.B., Benavides P.F.B., Müller B., De Toro G., Retamales J., Barajas O., Ahumada M., Morales E. (2019). ABCB1/4 Gallbladder Cancer Risk Variants Identified in India Also Show Strong Effects in Chileans. Cancer Epidemiol..

[34] Maechler M., Rousseeuw P., Croux C., Todorov V., Ruckstuhl A., Salibian-Barrera M., Verbeke T., Koller M., Eduardo L.T., Conceicao C. (2020). robustbase: Basic Robust Statistics R Package Version 0.93-6. http://CRAN.R-project.org/package=robustbaseGgplot2.

[35] Wickham H. (2016). ggplot2: Elegant Graphics for Data Analysis.

[36] Liu B., Chen Y., Yang J. (2016). LncRNAs are altered in lung squamous cell carcinoma and lung adenocarcinoma. Oncotarget.

[37] Gong W., Su Y., Liu Y., Sun P., Wang X. (2018). Long non-coding RNA Linc00662 promotes cell invasion and contributes to cancer stem cell-like phenotypes in lung cancer cells. J. Biochem..

[38] Liu Z., Yao Y., Huang S., Li L., Jiang B., Guo H., Lei W., Xiong J., Deng J. (2018). LINC00662 promotes gastric cancer cell growth by modulating the Hippo-YAP1 pathway. Biochem. Biophys. Res. Commun..

[39] He Y., Xu Y., Yu X., Sun Z., Guo W. (2021). The Vital Roles of LINC00662 in Human Cancers. Front. Cell Dev. Biol..

[40] Li J., Xue Y., Amin M.T., Yang Y., Yang J., Zhang W., Yang W., Niu X., Zhang H.Y., Gong J. (2019). ncRNA-eQTL: A database to systematically evaluate the effects of SNPs on non-coding RNA expression across cancer types. Nucleic Acids Res..

[41] Ruiz-Linares A., Adhikari K., Acuña-Alonzo V., Quinto-Sanchez M., Jaramillo C., Arias W., Fuentes M., Pizarro M., Everardo P., de Avila F. (2014). Admixture in Latin America: Geographic structure, phenotypic diversity and self-perception of ancestry based on 7342 individuals. PLoS Genet..

