# Supplementary material for: Identification of Circulating lncRNAs Associated with Gallbladder Cancer Risk by Tissue-Based Preselection, Cis-eQTL Validation, and Analysis of Association with Genotype-Based Expression
Source: Cancers (Basel). 2022 Jan 27;14(3):634. doi: 10.3390/cancers14030634 (PMC8833674; doi:10.3390/cancers14030634)
Supplement: Supplementary file 1 [file cancers-14-00634-s001.zip › cancers-1494658-supplementary.pdf]

Supplementary Materials: Identification of Circulating lncRNAs Associated with Gallbladder Cancer Risk by Tissue-Based Preselection, Cis-eQTL Validation, and Analysis of Association with Genotype-Based Expression

Figure S1. 39 high-quality preselected lncRNA candidates using ML, ordered by relative importance.

Figure S2. Predicted log 2 expression for *C22orf34* in the lncRNA-GBC association dataset.

Table S1. 36 preselected lncRNA candidates using J–T tests

Table S2. Identified and validated cis-lncRNA-eQTLs for the three preselected candidates.

Source code in R

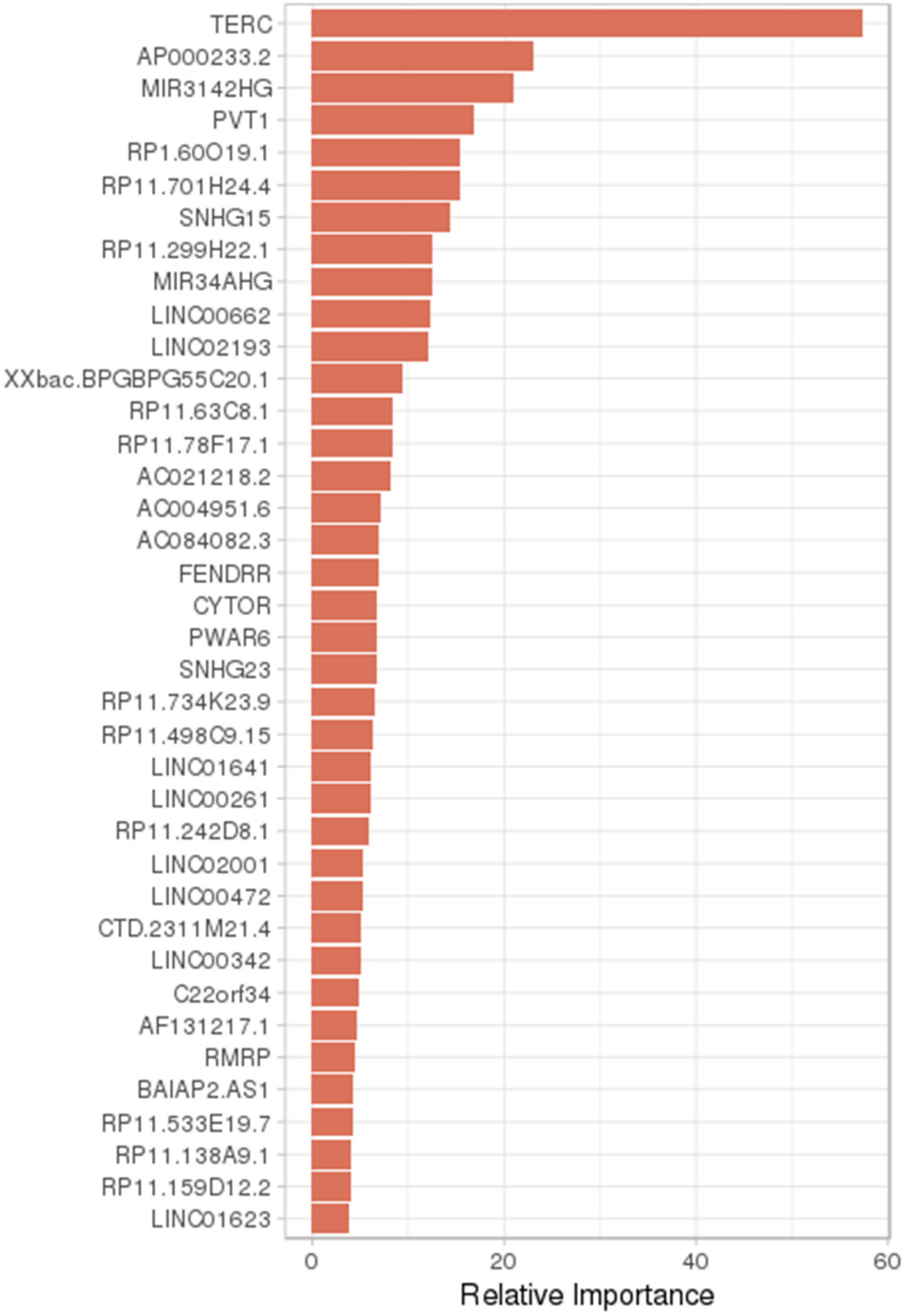

Figure S1. 39 high-quality preselected lncRNA candidates using ML, ordered by relative importance.

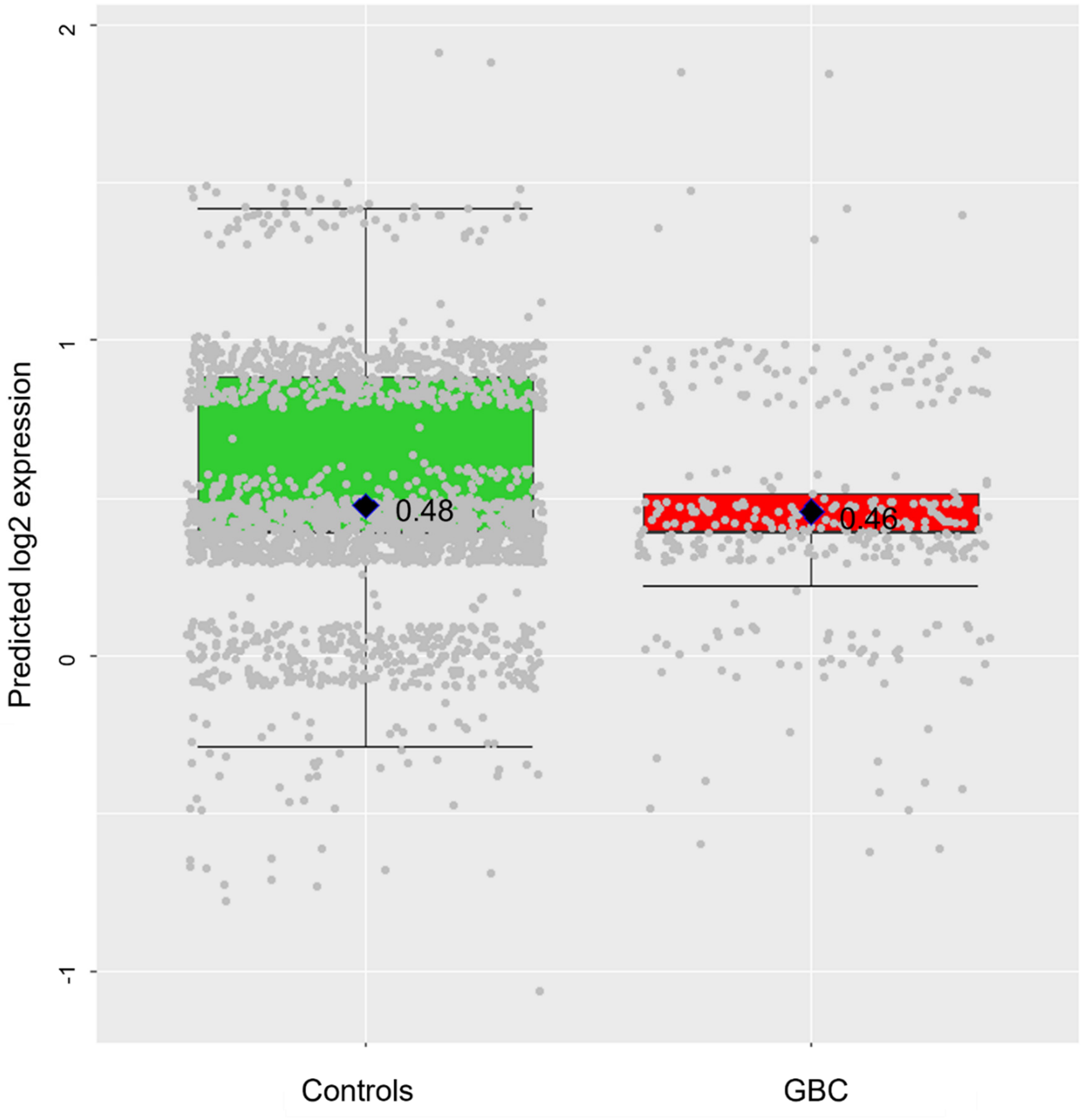

Figure S2. Predicted log 2 expression for *C22orf34* in the lncRNA-GBC association dataset.

Table S1. 36 preselected lncRNA candidates using J–T tests

lncRNA	adj. JT* *p*-value	Log2 expression in gallstone samples Median [5th to 95th]	OR† dysplasia Estimate [95%CI]	OR† GBC Estimate [95%CI]
AC084082.3	0.009	8.23 [1.45 - 9.93]	2.10 [0.86 - 5.11]	1.39 [1.04 - 1.85]
FAM95B1	0.009	1.44 [0.48 - 2.49]	0.15 [0.03 - 0.78]	0.13 [0.03 - 0.64]
HCG11	0.009	1.50 [0.65 - 2.78]	3.01 [1.14 - 7.99]	2.99 [1.12 - 7.96]
LINC00472	0.009	1.48 [0.63 - 2.53]	0.89 [0.43 - 1.88]	0.11 [0.02 - 0.64]
LINC00662	0.009	1.48 [0.55 - 4.38]	2.73 [1.41 - 5.30]	2.00 [1.12 - 3.58]
LINC00869	0.009	2.62 [0.92 - 3.97]	2.41 [1.19 - 4.85]	3.35 [1.48 - 7.56]
MIR155HG	0.009	7.66 [1.47 - 9.73]	1.55 [1.03 - 2.31]	2.33 [1.29 - 4.18]
MIR3142HG	0.009	10.56 [3.42 - 13.29]	1.31 [0.94 - 1.84]	3.14 [1.30 - 7.59]
PVT1	0.009	1.02 [0.45 - 1.75]	0.43 [0.10 - 1.86]	4.36 [0.87 - 21.86]
PWAR6	0.009	1.65 [0.85 - 3.37]	0.70 [0.33 - 1.47]	0.26 [0.08 - 0.80]
RP1.60O19.1	0.009	3.02 [0.92 - 5.32]	1.04 [0.67 - 1.64]	0.58 [0.34 – 1.00]
RP11.701H24.4	0.009	11.03 [1.47 - 12.47]	1.07 [0.87 - 1.32]	0.31 [0.11 - 0.84]
RP4.561L24.3	0.009	6.76 [1.44 - 8.87]	2.23 [1.24 - 4.03]	2.63 [1.30 - 5.34]
TERC	0.009	1.50 [0.73 - 2.85]	2.60 [1.17 - 5.78]	3.61 [1.53 - 8.55]
LL0XNC01.237H1.2	0.02	1.02 [0.45 - 1.96]	2.11 [0.90 - 4.93]	3.14 [1.13 - 8.73]
RP11.78F17.1	0.02	1.20 [0.50 - 1.82]	0.20 [0.04 - 0.98]	0.09 [0.02 - 0.52]
FENDRR	0.02	1.49 [0.82 - 2.88]	1.99 [0.75 - 5.26]	0.13 [0.02 - 0.71]
LINC00261	0.02	2.07 [0.54 - 4.41]	1.04 [0.64 - 1.67]	0.45 [0.22 - 0.90]
LINC02001	0.03	4.30 [1.20 - 6.60]	1.86 [1.21 - 2.86]	1.68 [1.12 - 2.50]
RP11.498C9.15	0.03	0.98 [0.46 - 1.59]	1.60 [0.58 - 4.45]	2.29 [0.73 - 7.15]
RP11.170M17.1	0.03	1.44 [0.45 - 4.27]	0.70 [0.38 - 1.29]	0.14 [0.02 - 0.77]
SNHG9	0.03	2.55 [1.09 - 4.33]	2.10 [1.07 - 4.13]	3.50 [1.43 - 8.60]
MEG3	0.03	3.69 [1.44 - 6.23]	0.95 [0.60 - 1.50]	0.39 [0.18 - 0.83]
RP6.74O6.2	0.03	1.46 [0.50 - 2.79]	0.77 [0.36 - 1.62]	0.51 [0.21 - 1.26]
RP1.140K8.5	0.04	1.49 [0.59 - 3.05]	1.02 [0.55 - 1.90]	0.34 [0.11 - 1.02]
RP11.304L19.13	0.04	1.44 [0.52 - 3.16]	1.43 [0.69 - 2.96]	2.77 [1.17 - 6.56]
CTD.2311M21.4	0.04	1.42 [0.45 - 2.79]	0.00 [0.00 - 0.15]	0.25 [0.06 - 1.02]
CTD.2626G11.2	0.04	1.44 [0.50 - 2.20]	0.17 [0.04 - 0.86]	0.42 [0.10 - 1.75]
OLMALINC	0.04	1.48 [0.51 - 2.91]	0.76 [0.35 - 1.64]	0.29 [0.09 - 0.95]
C22orf34	0.04	1.44 [0.48 - 3.68]	0.28 [0.08 - 1.07]	0.36 [0.10 - 1.28]
LINC00511	0.04	1.20 [0.46 - 1.90]	1.38 [0.4 - 4.75]	2.26 [0.76 - 6.77]
LINC00977	0.04	1.27 [0.52 - 2.29]	2.01 [0.75 - 5.38]	2.04 [0.81 - 5.13]
CTD.2210P24.2	0.04	1.46 [0.61 - 4.85]	0.85 [0.45 - 1.63]	2.52 [1.10 - 5.77]
MIR34AHG	0.04	6.35 [1.44 - 9.78]	1.60 [1.13 - 2.28]	2.02 [1.23 - 3.34]
CYTOR	0.04	1.44 [0.48 - 2.18]	0.85 [0.33 - 2.16]	2.27 [0.59 - 8.70]
RP11.714M23.2	0.04	1.44 [0.51 - 2.26]	0.91 [0.46 - 1.80]	0.36 [0.10 - 1.35]

*FDR-adjusted p value from J–T test.

†ORs were estimated using robust logistic regression models.

Table S2. Identified and validated cis-lncRNA-eQTLs for the three preselected candidates.

LINC00662

SNP ID	Chromosomal location (GRCh38)	MAF	Penetrance model	β1 coefficient	*p*-value β1	β2 coefficient	*p*-value β2
rs11083486	chr19:28407449:G:T	0.31	Additive	-0.74	0.01	-	-
rs11083486	chr19:28407449:G:T	0.31	Three-Genotypes	-0.96	0.03	-1.57	0.01
rs11083486	chr19:28407449:G:T	0.31	Dominant	-0.86	0.03	-	-
rs11083486	chr19:28407449:G:T	0.31	Recessive	1.29	0.03	-	-
rs142521755	chr19:27284894:T:A	0.07	Dominant	1.08	0.04	-	-

C22orf34

SNP ID	Chromosomal location (GRCh38)	MAF	Penetrance model	β1 coefficient	*p*-value β1	β2 coefficient	*p*-value β2
rs5770650	chr22:49683714:A:C	0.13	Additive	0.48	0.01	-	-
rs9628049	chr22:49551343:C:T	0.06	Additive	-0.60	0.02	-	-
rs5770650	chr22:49683714:A:C	0.13	Dominant	0.52	0.01	-	-
rs9628049	chr22:49551343:C:T	0.06	Dominant	-0.60	0.02	-	-
rs80641	chr22:49548950:G:T	0.11	Three-Genotypes	-2.19	0.006	-1.99	0.01
rs135786	chr22:49550809:G:A	0.12	Three-Genotypes	-2.25	0.004	-1.98	0.009
rs135787	chr22:49550871:G:A	0.12	Three-Genotypes	-2.25	0.004	-1.98	0.009
rs135788	chr22:49551103:T:G	0.12	Three-Genotypes	-2.25	0.004	-1.98	0.009
rs135789	chr22:49551309:T:C	0.12	Three-Genotypes	-2.25	0.004	-1.98	0.009
rs135791	chr22:49552575:C:T	0.12	Three-Genotypes	-2.25	0.004	-1.98	0.009
rs135792	chr22:49553166:G:C	0.12	Three-Genotypes	-2.25	0.004	-1.98	0.009
rs135793	chr22:49553257:G:A	0.12	Three-Genotypes	-2.25	0.004	-1.98	0.009
rs135794	chr22:49553508:T:C	0.12	Three-Genotypes	-2.25	0.004	-1.98	0.009
rs135796	chr22:49554141:A:G	0.12	Three-Genotypes	-2.25	0.004	-1.98	0.009
rs135797	chr22:49554220:G:C	0.12	Three-Genotypes	-2.25	0.004	-1.98	0.009
rs135798	chr22:49554437:A:G	0.12	Three-Genotypes	-2.25	0.004	-1.98	0.009
rs135799	chr22:49554674:G:A	0.12	Three-Genotypes	-2.25	0.004	-1.98	0.009
rs135800	chr22:49555086:C:T	0.12	Three-Genotypes	-2.25	0.004	-1.98	0.009
rs135801	chr22:49555128:G:A	0.12	Three-Genotypes	-2.25	0.004	-1.98	0.009
rs8140696	chr22:49555464:A:G	0.12	Three-Genotypes	-2.25	0.004	-1.98	0.009
rs8140728	chr22:49555542:A:G	0.12	Three-Genotypes	-2.25	0.004	-1.98	0.009
rs8140866	chr22:49555658:A:C	0.12	Three-Genotypes	-2.25	0.004	-1.98	0.009
rs1054180151	chr22:49555702:A:G	0.12	Three-Genotypes	-2.25	0.004	-1.98	0.009
rs135803	chr22:49555956:T:C	0.12	Three-Genotypes	-2.25	0.004	-1.98	0.009
rs135804	chr22:49556003:G:A	0.12	Three-Genotypes	-2.25	0.004	-1.98	0.009
rs135805	chr22:49556247:T:C	0.12	Three-Genotypes	-2.25	0.004	-1.98	0.009
rs135806	chr22:49556251:T:C	0.12	Three-Genotypes	-2.25	0.004	-1.98	0.009
rs135807	chr22:49556406:A:G	0.12	Three-Genotypes	-2.25	0.004	-1.98	0.009
rs135810	chr22:49557021:G:A	0.12	Three-Genotypes	-2.25	0.004	-1.98	0.009
rs135811	chr22:49557199:A:G	0.12	Three-Genotypes	-2.25	0.004	-1.98	0.009
rs135812	chr22:49557423:G:A	0.12	Three-Genotypes	-2.25	0.004	-1.98	0.009
rs135813	chr22:49557486:A:G	0.12	Three-Genotypes	-2.25	0.004	-1.98	0.009
rs135814	chr22:49557526:T:C	0.12	Three-Genotypes	-2.25	0.004	-1.98	0.009
rs9627745	chr22:49557770:C:G	0.12	Three-Genotypes	-2.25	0.004	-1.98	0.009

rs35356406	chr22:49558924:G:C	0.12	Three-Genotypes	-2.25	0.004	-1.98	0.009
rs135815	chr22:49559001:T:C	0.12	Three-Genotypes	-2.25	0.004	-1.98	0.009
rs135816	chr22:49559524:C:T	0.12	Three-Genotypes	-2.25	0.004	-1.98	0.009
rs135817	chr22:49560766:G:A	0.12	Three-Genotypes	-2.25	0.004	-1.98	0.009
rs135821	chr22:49562360:T:G	0.12	Three-Genotypes	-2.25	0.004	-1.98	0.009
rs13055340	chr22:49562667:T:C	0.12	Three-Genotypes	-2.25	0.004	-1.98	0.009
rs1661563636	chr22:49562872:C:T	0.12	Three-Genotypes	-2.25	0.004	-1.98	0.009
rs112515352	chr22:49563159:G:A	0.12	Three-Genotypes	-2.25	0.004	-1.98	0.009
rs135822	chr22:49563851:T:C	0.12	Three-Genotypes	-2.25	0.004	-1.98	0.009
rs135823	chr22:49564023:G:A	0.12	Three-Genotypes	-2.25	0.004	-1.98	0.009
rs135826	chr22:49565810:G:A	0.11	Three-Genotypes	-2.25	0.004	-1.98	0.009
rs6009823	chr22:49692686:C:T	0.12	Three-Genotypes	1.58	0.004	0.96	0.04
rs6009824	chr22:49692725:G:A	0.12	Three-Genotypes	1.58	0.004	0.96	0.04

Source code in R

```
##############################################################################
#
# program name:         01_LINC00662_preselection.R
# program title:        Preselection of differentially expressed lncRNAs
#                       along the sequence GS -> Dys -> GBC
# author:               Alice Blandino
# version:              1.0
# date:                 15.11.2021
#
# description:          Calculation of two-sided Jonckheere-Terpstra test
#
# input files:          01_data_LINC00662_preselection.txt
#
# Available at          www.biometrie.uni-heidelberg.de/
#                       StatisticalGenetics/Software_and_Data
#
##############################################################################

# "01_data_LINC00662_preselection.txt"
#
# A text file with a header line, and then one line per study participant
# with the following two fields:
#
# LINC00662             expression of LINC00662 in FFPE tissue
# group                 patients' status (gallstones,dysplasia,GBC)

# install and activate package to run two-sided J-T test
install.packages("DescTools", dependencies = TRUE)
library(DescTools)
# load data of study participants
setwd("*Path:\*")
data_preselection <- read.table("01_data_LINC00662_preselection.txt", header=T)
# order the group variable
data_preselection$group <- factor(data_preselection$group,
                                levels=c("GBC", "dysplasia", "gallstones"),
                                ordered=TRUE)
# perform J-T test
jt.test<-JonckheereTerpstraTest(data_preselection$LINC00662,
                              data_preselection$group,
                              alternative = "two.sided",nperm = 5000)
```

```
################################################################################
#
# program name:          02_LINC00662_validation.R
# program title:         Selection of best model for prediction
# author:                Alice Blandino
# version:               1.0
# date:                  15.11.2021
#
# description:           Model selection based on robust AIC from robust
#                        linear regression models
# input files:           02_data_LINC00662_validation.txt
# Available at           www.biometrie.uni-heidelberg.de/
#                        StatisticalGenetics/Software_and_Data
#
################################################################################

# "02_data_LINC00662_validation.txt"
#
# A text file with a header line, and then one line per study participant
# with the following two fields:
#
# LINC00662              LINC00662 expression in serum
# rs11083486             genotype for rs11083486 (0=G/G ;1=G/T ;2=T/T)
# rs142521755            genotype for rs142521755 (0=A/A ;1=A/T ;2=T/T)
# age                    study participants' age
# gender                 study participants' gender
# PC1-PC10               first 10 PCs

# install and activate package to add variables to dataframe
install.packages("dplyr", dependencies = TRUE)
library(dplyr)

setwd("*Path:\*")
data_validation <- read.table("02_data_LINC00662_validation.txt", header=T)

# add new variables where:
# rs11083486 is once encoded dominantly (0+1 vs. 2), once encoded recessively (0 vs. 1+2)
# rs142521755 is encoded dominantly (0+1 vs. 2)
data_validation_new<-data_validation%>%
            mutate(rs11083486.dominant=ifelse(rs11083486=="0",1,rs11083486),
               rs11083486.recessive=ifelse(rs11083486=="2",1,rs11083486),
               rs142521755.dominant=ifelse(rs142521755=="0",1,rs142521755))

# model selection
# install and activate package to run robust linear regression models
install.packages(c("MASS","clickR",”AICcmodavg”), dependencies = TRUE)
library(MASS)
library(clickR)
library(AICcmodavg)
# 1.
# MODELS WITH rs11083486 ONLY
# additive
model.rs11083486.additive<-
rlm(LINC00662~rs11083486+age+gender+PC1+PC2+PC3+PC4+PC5+PC6+PC7+PC8+PC9+PC10,data=data_validati
on_new)
# three-genotypes
model.rs11083486.three<-
rlm(LINC00662~as.factor(rs11083486)+age+gender+PC1+PC2+PC3+PC4+PC5+PC6+PC7+PC8+PC9+PC10,data=da
ta_validation_new)
# dominant
```

```
model.rs11083486.dom<-
rlm(LINC00662~rs11083486.dominant+age+gender+PC1+PC2+PC3+PC4+PC5+PC6+PC7+PC8+PC9+PC10,data=dat
a_validation_new)
# recessive
model.rs11083486.rec<-
rlm(LINC00662~rs11083486.recessive+age+gender+PC1+PC2+PC3+PC4+PC5+PC6+PC7+PC8+PC9+PC10,data=dat
a_validation_new)

# 2.
# MODEL WITH rs142521755 ONLY
# rs142521755 dominant
model.rs142521755.dom<-
rlm(LINC00662~rs142521755.dominant+age+gender+PC1+PC2+PC3+PC4+PC5+PC6+PC7+PC8+PC9+PC10,data=d
ata_validation_new)

# 3.
# MODELS WITH BOTH rs11083486 AND rs142521755
# rs11083486 additive & rs142521755 dominant
model.add.dom<-
rlm(LINC00662~rs11083486+rs142521755.dominant+age+gender+PC1+PC2+PC3+PC4+PC5+PC6+PC7+PC8+PC9+
PC10,data=data_validation_new)
# rs11083486 three-genotypes & rs142521755 dominant
model.three.dom<-
rlm(LINC00662~as.factor(rs11083486)+rs142521755.dominant+age+gender+PC1+PC2+PC3+PC4+PC5+PC6+PC7+P
C8+PC9+PC10,data=data_validation_new)
# rs11083486 dominant & rs142521755 dominant
model.dom.dom<-
rlm(LINC00662~rs11083486.dominant+rs142521755.dominant+age+gender+PC1+PC2+PC3+PC4+PC5+PC6+PC7+P
C8+PC9+PC10,data=data_validation_new)
# rs11083486 recessive & rs142521755 dominant
model.rec.dom<-
rlm(LINC00662~rs11083486.recessive+rs142521755.dominant+age+gender+PC1+PC2+PC3+PC4+PC5+PC6+PC7+P
C8+PC9+PC10,data=data_validation_new)

# create a dataframe with each model's name and its AIC
# vector with AICs:
AICs<-
c(AIC(model.rs11083486.additive),AIC(model.rs11083486.three),AIC(model.rs11083486.dom),AIC(model.rs1108348
6.rec),

AIC(model.rs142521755.dom),AIC(model.add.dom),AIC(model.three.dom),AIC(model.dom.dom),AIC(model.rec.dom
))
# vector with models' characteristics
models<-c("rs11083486.additive","rs11083486.three","rs11083486.dominant","rs11083486.recessive",
        "rs142521755.dominant","additive+dominant","three+dominant","dominant+dominant","recessive+dominant")
# dataframe with both AIC and models' characteristics
summary.AIC<-data.frame(AICs,models)
# find which model has the lowest RAIC
summary.AIC[order(summary.AIC$AICs),,drop=FALSE] [1,]
```

```
##############################################################################
#
# program name:         03_LINC00662_prediction.R
# program title:        genotype-based lncRNA expression prediction
# author:               Alice Blandino
# version:              1.0
# date:                 15.11.2021
#
# description:          prediction of lncRNA based on individual genotype data
#                       and quantification of GBC risk associated to it
# input files:          03_data_LINC00662_prediction.txt
# Available at          www.biometrie.uni-heidelberg.de/
#                       StatisticalGenetics/Software_and_Data
#
##############################################################################

# "03_data_LINC00662_prediction.txt"
#
# A text file with a header line, and then one line per study participant
# with the following two fields:
#
# rs11083486            genotype for rs11083486 (0=T/T ;1=G/T ;2=G/G)
# rs142521755           genotype for rs142521755 (0=A/A ;1=A/T ;2=T/T)
# pheno                 patients' status (Control, Case)
# age                   study participants' age
# gender                study participants' gender
# PC1-PC10              first 10 PCs

# install and activate package to add variables to dataframe
install.packages(c("robustbase","dplyr"), dependencies = TRUE)
library(robustbase)
library(dplyr)

setwd("*Path:\*")
data_prediction <- read.table("03_data_LINC00662_prediction.txt", header=T)

# calculate the SNP-based expression
data_prediction_calculation<-data_prediction%>%
  mutate(rs11083486.coeff=ifelse(rs11083486=="0",-0.7352*0,ifelse(rs11083486=="1",-0.7352*1,-0.7352*2)),
      rs142521755.coeff=ifelse(rs142521755=="0",1.0797*0,ifelse(rs142521755=="1",1.0797*0,1.0797)),
      predicted.LINC00662=0.9267+rs11083486.coeff+rs142521755.coeff)

# association analysis fitting robust logistic regression model

# set controls as baseline category
data_prediction_calculation$pheno<-ordered(data_prediction_calculation$pheno, levels = c("Control", "Case"))

# model fitting
mod<-
glmrob(as.factor(data_prediction_calculation$pheno)~predicted.LINC00662+age+gender+PC1+PC2+PC3+PC4+PC5+
PC6+PC7+PC8+PC9+PC10,family = binomial, method= "Mqle",control=
glmrobMqle.control(tcc=1.2),data=data_prediction_calculation)
summary(mod)

# extract Oddsratio for Cases
exp(summary(mod)$coefficients[2])

# extract lower and upper limits for confidence intervals
exp(summary(mod)$coefficients[2] + qnorm(c(0.5,0.025,0.975)) * summary(mod)$coefficients[2,2])[2]
exp(summary(mod)$coefficients[2] + qnorm(c(0.5,0.025,0.975)) * summary(mod)$coefficients[2,2])[3]
```

```
################################################################################
#
# program name:        04_ML_models.R
# program title:       XGBoost algorithm
# author:              Sinan U. Umu
# version:             1.0
# date:                15.11.2021
#
# description:         Extreme gradient boosting (XGBoost) algorithm
#                      to train three-class classification ML models
#
################################################################################

require(xgboost)
require(parallel)
require(doParallel)
require(tidyverse)
require(h2o)

setwd("*Path:\*")
lcrna_gbc=openxlsx::read.xlsx("lncRNAs_normalized_filtered.xlsx") %>% filter(!ID %in% excluded_samples)

h2o.init(nthreads = 15,max_mem_size = "200G")

function_h2o_data_split=function(lcrna_gbc,ratio=0.70,seed=1) { #seed=1

  dysplasia=h2o.splitFrame(as.h2o(lcrna_gbc  %>% mutate(group=factor(group)) %>% filter(group == "dysplasia"))
,ratios = ratio,seed = seed)
  gallstones=h2o.splitFrame(as.h2o(lcrna_gbc  %>% mutate(group=factor(group)) %>% filter(group == "gallstones"))
,ratios = ratio,seed = seed)

  GBC=h2o.splitFrame(as.h2o(lcrna_gbc  %>% mutate(group=factor(group))%>% filter(group == "GBC")) ,ratios =
ratio,seed = seed)

  train_x=c(dysplasia[[1]],gallstones[[1]],GBC[[1]]) %>% purrr::map(~as_tibble(.)) %>% bind_rows()
  test_x=c(dysplasia[[2]],gallstones[[2]],GBC[[2]]) %>% purrr::map(~as_tibble(.)) %>% bind_rows()




  return(list(as.h2o(train_x),as.h2o(test_x)))
}


lncrna_split=function_h2o_data_split(lcrna_gbc,seed=1) #create the datasets training, validation

train_x=as.h2o(upSample(as.data.frame(lncrna_split[[1]]),as.data.frame(lncrna_split[[1]]) %>% pull(group))) #training
dataset
test_x=lncrna_split[[2]] #validation dataset

y=c("group")
x =setdiff(names(train_x), c(y,"Class","age","sex","ID")) #predictors, only lncRNAs left




hyper_params <- list(ntrees = seq(10, 300, 1),
            learn_rate = seq(0.005, 0.3, 0.01),
            max_depth = seq(1, 7, 1),
            sample_rate = seq(0.1, 1.0, 0.01),
            col_sample_rate = seq(0.2, 1.0, 0.01),
            reg_alpha=seq(0,0.3,0.025),
```

```
            gamma=seq(3,7,1),
            reg_lambda=seq(0,0.3,0.025)

)



search_criteria <- list(strategy = "RandomDiscrete",
             max_models = 50
)

#grid search for hyperparamater tuning via crossvalidation
xgb_grid <- h2o.grid(algorithm = "xgboost",
             grid_id = "xgboostgbc_grid_random_seed8",
             x = x, y = y,
             training_frame = train_x,
             #validation_frame=test_x,
             #reg_lambda=0,

             auc_type = "WEIGHTED_OVR",

             #validation_frame=test_x,
             nfolds = 5,
             seed = 8,
             #stopping_metric="mean_per_class_error",
             #stopping_tolerance=0.01,
             #stopping_rounds=10,

             hyper_params = hyper_params_hard_code,
             search_criteria = search_criteria)

best_model <- h2o.getModel(xgb_grid@model_ids[[1]])



## extract best variables
as.tibble(h2o.varimp(best_model)) %>% dplyr::mutate(median=median(relative_importance)) %>%
dplyr::mutate(f=ifelse(relative_importance >= median,"PASS","FAIL"))

##train the best using the hyperparamaters on the training dataset

function_get_model_params=function(best_model,param_name) {

  best_model@allparameters[[param_name]]
}


#train the best using the hyperparamaters
test_model <- h2o.xgboost(x = x,
              y = y,
              training_frame = train_x,


              booster= function_get_model_params(best_model,"booster"),

              normalize_type=function_get_model_params(best_model,"normalize_type"),
              seed = 8,

              ntrees = function_get_model_params(best_model,"ntrees"),
              #nround=function_get_model_params(best_model,"nround"),
              learn_rate = function_get_model_params(best_model,"learn_rate"),
```

```
                    eta=function_get_model_params(best_model,"eta"),
                    max_depth = function_get_model_params(best_model,"max_depth"),
                    sample_rate = function_get_model_params(best_model,"sample_rate"),
                    subsample = function_get_model_params(best_model,"subsample"),
                    col_sample_rate = function_get_model_params(best_model,"col_sample_rate"),
                    colsample_bylevel=function_get_model_params(best_model,"colsample_bylevel"),
                    reg_alpha=function_get_model_params(best_model,"reg_alpha"),
                    reg_lambda=function_get_model_params(best_model,"reg_lambda"),
                    colsample_bytree=function_get_model_params(best_model,"colsample_bytree"),
                    col_sample_rate_per_tree=function_get_model_params(best_model,"col_sample_rate_per_tree"),
                    colsample_bynode=function_get_model_params(best_model,"colsample_bynode"),
                    gamma=function_get_model_params(best_model,"gamma"),
                    min_split_improvement=function_get_model_params(best_model,"min_split_improvement"),
                    gainslift_bins=-1,
                    grow_policy="depthwise",
                    fold_assignment="Random",
                    tweedie_power=1.5,
                    max_leaves=0,
                    max_bins=function_get_model_params(best_model,"max_bins"),
                    min_rows=function_get_model_params(best_model,"min_rows"),
                    min_child_weight=function_get_model_params(best_model,"min_child_weight"),
                    #stopping_tolerance=0.01,
                    auc_type = "WEIGHTED_OVR",
                    nfolds=5
)


#get model performance on the test dataset (this is reported in the manuscript)
h2o.performance(test_model,test_x)


#these are the most important hyperparamaters, found via crossvalidation
hyper_params_found_by_the_best_model <- list(ntrees = 245,
                                  learn_rate = 0.115,
                                  max_depth = 6,
                                  sample_rate = 0.66,
                                  col_sample_rate = 0.47,
                                  reg_alpha=0,
                                  gamma=3,
                                  reg_lambda=0.3

)
```
